# Difference in postprandial GLP-1 response despite similar glucose kinetics after consumption of wheat breads with different particle size in healthy men

**DOI:** 10.1007/s00394-016-1156-6

**Published:** 2016-02-08

**Authors:** Coby Eelderink, Martijn W. J. Noort, Nesli Sozer, Martijn Koehorst, Jens J. Holst, Carolyn F. Deacon, Jens F. Rehfeld, Kaisa Poutanen, Roel J. Vonk, Lizette Oudhuis, Marion G. Priebe

**Affiliations:** 1 Center for Medical Biomics, University Medical Center Groningen, University of Groningen, Hanzeplein 1, 9713 GZ Groningen, The Netherlands; 2 Top Institute Food and Nutrition, Wageningen, The Netherlands; 3 TNO Food and Nutrition, Zeist, The Netherlands; 4 VTT Technical Research Centre of Finland, Espoo, Finland; 5 Department of Laboratory Medicine, Center for Liver Digestive and Metabolic Diseases, University Medical Center Groningen, University of Groningen, Groningen, The Netherlands; 6 NNF Center for Basic Metabolic Research, Department of Biomedical Sciences, The Panum Institute, University of Copenhagen, Copenhagen, Denmark; 7 Department of Clinical Biochemistry, Rigshospitalet, University of Copenhagen, Copenhagen, Denmark

**Keywords:** Glucagon-like peptide-1, Glycemic index, Glucose kinetics, Bile acids, Wheat kernels, Bread processing

## Abstract

**Purpose:**

Underlying mechanisms of the beneficial health effects of low glycemic index starchy foods are not fully elucidated yet. We varied the wheat particle size to obtain fiber-rich breads with a high and low glycemic response and investigated the differences in postprandial glucose kinetics and metabolic response after their consumption.

**Methods:**

Ten healthy male volunteers participated in a randomized, crossover study, consuming ^13^C-enriched breads with different structures; a control bread (CB) made from wheat flour combined with wheat bran, and a kernel bread (KB) where 85 % of flour was substituted with broken wheat kernels. The structure of the breads was characterized extensively. The use of stable isotopes enabled calculation of glucose kinetics: rate of appearance of exogenous glucose, endogenous glucose production, and glucose clearance rate. Additionally, postprandial plasma concentrations of glucose, insulin, glucagon, incretins, cholecystokinin, and bile acids were analyzed.

**Results:**

Despite the attempt to obtain a bread with a low glycemic response by replacing flour by broken kernels, the glycemic response and glucose kinetics were quite similar after consumption of CB and KB. Interestingly, the glucagon-like peptide-1 (GLP-1) response was much lower after KB compared to CB (iAUC, *P* < 0.005). A clear postprandial increase in plasma conjugated bile acids was observed after both meals.

**Conclusions:**

Substitution of 85 % wheat flour by broken kernels in bread did not result in a difference in glucose response and kinetics, but in a pronounced difference in GLP-1 response. Thus, changing the processing conditions of wheat for baking bread can influence the metabolic response beyond glycemia and may therefore influence health.

**Electronic supplementary material:**

The online version of this article (doi:10.1007/s00394-016-1156-6) contains supplementary material, which is available to authorized users.

## Introduction

Consumption of foods with a low glycemic index (GI) instead of those with a high GI is considered beneficial for health, being associated with a decreased risk for the development of obesity, insulin resistance, and type 2 diabetes (T2DM) [1–5], although this association is not always found [6]. Based on results from a prospective cohort study, it was suggested that replacement of high GI bread by low GI bread in the diet may reduce the risk of developing T2DM [7]. Possible explanations were, for instance, that consumption of a low GI diet might be associated with less weight gain [4] or decreased development of β-cell failure and insulin resistance [1, 3]. However, the underlying mechanisms responsible for the beneficial effects need further study.

The postprandial glycemic response obviously depends on intestinal glucose influx, but is also influenced by suppression of endogenous glucose production (EGP) and increased glucose uptake in tissues, processes that are mainly regulated by the pancreatic hormones insulin and glucagon. The incretin hormones glucose-dependent insulinotropic polypeptide (GIP) and glucagon-like peptide-1 (GLP-1), released postprandial from intestinal K and L cells, respectively, are known to potentiate the insulin response to a carbohydrate-rich meal and are therefore important factors in glucose metabolism. Besides their role as incretin hormones, GIP is involved in fat metabolism [8], and GLP-1 is involved in decreasing gastric emptying rate [9], suppression of glucagon [10], as well as increasing satiety [11], and, in rodents, preserving β-cell function [12]. Nowadays, bile acids (BAs) are also being recognized as signaling molecules in glucose metabolism. For instance, overexpression of the BA receptor TGR5 [13] and administration of taurocholic acid (TCA) [14] in mice was associated with elevated GLP-1 concentrations in response to an oral glucose tolerance test (OGTT). Although BAs are mainly released in response to fat ingestion, an increase in several plasma conjugated BAs was found after an OGTT [15, 16]. Thus, BAs, as well as (indirectly) the gastrointestinal hormone cholecystokinin (CCK) which stimulates gall bladder contraction, might also play a role in glucose metabolism after consumption of a carbohydrate-rich meal.

We were, therefore, interested in studying both the postprandial glucose kinetics and the metabolic responses to bread with a high and a low glycemic response, using wheat particle size as a variable factor. Postprandial glucose responses to wheat products prepared with different flour or grain particle size have been shown to decrease with increasing particle size [17], likely explained by decreasing the rate of amylolysis. Replacement of finely ground wheat flour by increasing the proportion of cracked wheat in bread (to 50 and 75 %) also resulted in a reduction in GI [18].

In this crossover study, we investigated the metabolic effects of consumption of two ^13^C-labeled wheat breads, with the same overall composition, only varying in structure; a bread prepared with flour and 85 % broken wheat kernels and a control bread made from wheat flour combined with wheat bran, to obtain a similar dietary fiber content in both breads. We hypothesized that, due to the variation in particle size, the glycemic response, the underlying glucose kinetics, and possibly other factors involved in glucose metabolism would be different between both breads.

## Subjects and methods

### Subjects

Ten healthy men [age 24 ± 0.6 years, BMI 22 ± 0.2 kg/m^2^ (mean ± SEM)] were recruited. The main criteria for exclusion were use of medication, blood donation, or use of antibiotics in the past three months, gastrointestinal surgery or dysfunction, inflammatory diseases, and diabetes mellitus. Approval was obtained from the Medical Ethics Committee of the ‘Beoordeling Ethiek Biomedisch Onderzoek’ foundation, Assen, The Netherlands. Each subject gave written informed consent for the study. This trial was registered at trialregister.nl as NTR3020.

### Experimental design

In the overall study design, addressing two different research questions, four different wheat-based products were tested. To increase clarity and be able to focus on the results of one of these questions, only two meals are described in this paper. A description of the other products and the results can be found elsewhere [19]. The study was performed in a randomized, crossover manner, with at least 1 week between each study day. The subjects were asked to refrain from consuming foods naturally high in ^13^C, like cane sugar, corn products, and pineapple, for 3 days preceding the experiments and from alcohol consumption and strenuous exercise for 24 h before each study day. Food intake on the day before each study day was individually standardized. To minimize variation, a standard evening meal was provided at the commercial research facility (QPS Netherlands B.V.), where the participants stayed overnight. In the evening, a venous catheter was inserted in each forearm for blood collection and for infusion of the tracer D-[6,6-^2^H_2_]glucose (98 % ^2^H atom percent excess, Isotec) [20, 21]. Subjects fasted overnight, but were allowed to drink water. In the morning (*t* = −122 min), a bolus of 26.7 mL D-[6,6-^2^H_2_]glucose solution (80 × 0.07 mg/kg body weight) was injected within 2 min, and a continuous infusion of 0.07 mg/kg body weight D-[6,6-^2^H_2_]glucose per min was started (*t* = −120 min) and maintained for 8 h (until *t* = 360 min). The bolus amount was 80× the infusion rate over 1 min, according to the paper of Tissot et al [20]. The infusion rate over 1 min was 0.07 mg/kg BW [21], to reach a good steady state. So the continuous infusion contained 4.2 mg/kg BW per 60 min, administered at a desired infusion rate of 20 mL/h, resulting in the concentration of 0.21 mg/mL/kg BW. The bolus had the same concentration, and therefore, to administer 80 × 0.07 mg/kg BW an amount of 26.7 mL was administered.

**Fig. 1:**
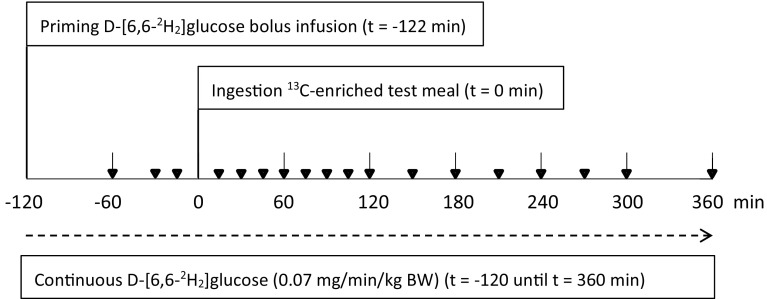
Simplified schematic time line of each study day. Blood was collected at 18 time points, indicated by *inverted triangle*. Also, several breath and urine samples were collected during each study day

Two hours after the start of the infusion the test meal was ingested (*t* = 0 min). Figure 1 shows a simplified time line of each study day. Water (150 mL) was provided hourly, starting at *t* = 120 min. During the study period physical activity was limited.

### Test meals

Two types of wheat bread were prepared (TNO, Zeist, The Netherlands), with the same composition, but a different structure, due to different milling and bread making processes; a kernel bread (KB) prepared with flour and 85 % broken wheat kernels, and a control bread (CB) made from wheat flour, combined with wheat bran, to obtain the same dietary fiber content as in the KB.

To provide the necessary ingredients, unlabeled (1.085 at.% ^13^C) wheat grains [T. *aestivum* var *Capo*, grown in Austria] were milled in different ways; by conventional roller-milling the wheat kernels were milled to obtain refined white flour and wheat bran, and broken wheat kernels were obtained using a breaker mill. For ^13^C-enrichment of the products, ^13^C-labeled wheat [*T. aestivum* var *Paragon* (1.359 at.% ^13^C)], cultured in a ^13^CO_2_ enriched atmosphere, was used. A small part of the ^13^C-labeled wheat kernels was milled to obtain a fine wholemeal wheat flour, and the other part was broken to obtain broken kernels that were similar to the unlabeled broken kernels.

CB was prepared with 1446 g unlabeled white wheat flour, 240 g ^13^C-labeled wholemeal wheat flour, 314 g wheat bran, 1300 g water, 33.4 g yeast, 36 g salt, 3 g malt, and 70 ppm ascorbic acid. After kneading, the dough was left to rise for 30 min, molded, and left to rise for 60 min. KB was prepared with 264 g unlabeled white wheat flour, 36 g ^13^C-labeled wholemeal wheat flour, 1496 g unlabeled broken wheat kernels, and 204 g ^13^C-labeled broken wheat kernels. All broken kernels were soaked in 1000 g water overnight at 10 °C. The following day, the soaked kernels were mixed with the flour, 230 g water, 33.4 g yeast, 36 g salt, 3 g malt, and 70 ppm ascorbic acid. The resulting dough was left to rise for 30 min, molded, and left to rise for 50 min. Subsequently, the breads were baked for 30 min at 240 °C. Bread slices were stored at −20 °C until use.

All test meals provided 50 g available carbohydrates; for the CB, the portion size was 138 g and for the KB 137 g. The breads were consumed together with 10 g light margarine (4 g fat), 2 slices lean ham (5 g fat, 6 g protein), and 250 mL tap water within 20 min. We assume that the digestibility of starch from both test products is similarly affected by this addition.

### Bread characterization

Starch, dietary fiber, and moisture content were determined at Eurofins Analytico Food, The Netherlands. To quantify starch fractions (G_T_, G_RA_, G_SA_, G_TA_, and RS) of the test meals in vitro, an adapted version of the Englyst method [22] was used [23].

Particle size distribution of the broken kernels was determined by sieve analysis in duplicate.

Bread products volume (mL) was determined in triplicate by rapeseed displacement (AACCI Method 10-05.01 [24]) and, together with the bread weight (g), the product overall specific volume (mL/g) and density (g/mL) were obtained.

Breads were further characterized using microscopy and X-ray microtomography (XRT, porosity). A detailed description of both techniques can be found in Online Resource 1.

### Sample collection

Blood was collected into several blood collection tubes (BD Diagnostics): 2 mL fluoride tubes (NaF) for glucose and bile acid measurements, 3 mL EDTA tubes [+30 µL DPP-4 inhibitor (Millipore)] for GIP, GLP-1, glucagon, and insulin determination, and 3 mL lithium/heparin tubes (CCK). Three basal blood samples were collected (*t* = −60, −30, −15 min), and postprandial samples were drawn every 15 min for 2 h, every 30 min for an additional 3 h, and once after 6 h. To obtain heparin plasma, blood was collected at *t* = −60, *t* = −15 min and then every 30 min for the first 3 h, and hourly for the last 3 h. After centrifugation (1300×*g* for 10 min at 4 °C), plasma aliquots were stored at −20 °C (NaF and EDTA plasma) or −80 °C (heparin plasma) until analysis.

Breath samples were collected by breathing through a straw into 10 mL Exetainer^®^ vials (Labco Limited). Two basal breath samples were collected (*t* = −30, *t* = −5 min), and after the test meal, a sample was taken every 30 min until *t* = 360 min.

To get an impression about the impact of the breads on appetite, subjects were asked to rate their feeling of appetite (hunger) using a visual analog scale (VAS) at 15 min before and hourly after the test meal, although the number of subjects (*n* = 10) is insufficient to detect differences in these subjective measures [25]. At the same time points, their feeling and extent of discomfort (abdominal pain, flatulence, other complaints) was recorded (0 = no complaints, 3 = severe complaints). Shortly after consumption of the meal, the subjects scored (VAS) how tasty the meal was.

### Measurement of plasma concentrations

Plasma glucose concentrations were measured on a Roche/Hitachi Modular automatic analyzer (Roche Diagnostics, Hitachi) using a glucose hexokinase method. The within- and between-run CV were ≤2 %. The ARCHITECT^**®**^ insulin assay (Abbott Laboratories) was used to determine insulin concentrations in plasma. The total CV of this chemiluminescent microparticle immunoassay was ≤7 %. The glucagon assay was directed against the C-terminal of the glucagon molecule (antibody code no. 4305) and therefore measures glucagon of mainly pancreatic origin [26]. Total GIP was measured using the C-terminally directed antiserum (no. 80867) [27], which reacts fully with intact GIP (1-42) and the N-terminally truncated metabolite GIP (3-42). Total GLP-1 concentrations were determined as previously described [28], using a radioimmunoassay (antiserum no. 89390) specific for the C-terminal of the GLP-1 molecule and reacting equally with intact GLP-1 and the primary (N-terminally truncated) metabolite. The glucagon and incretin assays have detection limits of <2 pmol/L, and an intra-assay coefficient of variation of approximately 6 %. CCK was measured using antiserum no. 92128, which binds the bioactive forms of CCK with equal potency without cross-reactivity with any gastrin [29]. The detection limit of the CCK assay is 0.1 pmol/L, and the intra-assay coefficient of variation approximately 5 %.

### Measurement of plasma bile acids

Fasting and postprandial concentrations of 15 individual BAs were determined using LC/MS: the primary BAs [cholic acid (CA) and chenodeoxycholic acid (CDCA)], the secondary BAs [deoxycholic acid (DCA), ursodeoxycholic acid (UDCA), and lithocholic acid (LCA)], as well as their glycine (G, glyco-) and taurine (T, tauro-) conjugates. The lower limit of quantitation (LOQ) was 0.05 µM. Concentrations of TCA, LCA, GLCA, TLCA, TDCA, UDCA, and TUDCA were below the LOQ. The intra- and inter-assay CV ranged from 1.6 to 11.3 and 4.4 to 13.3 %, respectively. For sample preparation, 250 µl of internal standard solution was mixed with 25 µl plasma and centrifuged at 15.900×*g* for 10 min. The supernatant was transferred into a new vial, evaporated under nitrogen at 40 °C, and reconstituted in 100 µl of 50 % methanol. The solution was filtered with a 0.2-µm centrifugal filter at 2000×*g* for 10 min. After this step, the samples (injection volume 10 µl) were ready for analysis, using two different LC/MS systems. A detailed description of the systems and settings can be found in Online Resource 2.

### Analysis of isotopic enrichment (breath and plasma) and calculations

Analysis of ^13^C abundance in breath CO_2_ was performed using GC/IRMS (Delta Plus XL; Thermo Fisher Scientific) measuring the ^13^C/^12^C ratio versus the international standard Pee Dee Belemnite (δ^13^C_PDB_, in ‰).

Plasma sample preparation required for analysis of isotopic enrichment by GC (derivatization) is described in detail elsewhere [30, 31]. ^2^H enrichment was measured by GC/MS as previously described [30], and ^13^C/^12^C isotope ratio was measured using GC/C/IRMS as previously described [32], both with some modifications [33].

Calculation of glucose kinetics was performed as previously reported [34].

### Incremental areas under the curve (iAUC)

To determine differences in glucose kinetics and plasma glucose, insulin, glucagon, incretin, CCK, and bile acid concentrations, the 0–2 and 0–6 h iAUCs were calculated as previously described [34].

### Statistics

Human data are presented as mean ± SEM, *n* = 10. Baseline-subtracted values are shown in the figures and were used in the analyses as well, for clarity, consistency, and comparison of variables. The overall study involved testing of four test meals. From the statistical analysis of the full experiment, we highlight only the comparisons involving the products of current interest. For parameters that result in individual points of a time curve, we fitted a model that accounts for the differences between the 10 subjects and the four occasions, the main effects of treatment and time, respectively, and the time × treatment interaction. Residual plots based on such a model were used to assess approximate normality and homogeneity of variances. Parameters that were not compatible with these assumptions were transformed before definitive analysis. Most variables were log-transformed, except for glucose (reciprocal values), VAS scores (angular transformation), RaE, and ^13^CO_2_ values (Poisson regression model on log scale).

We performed *F* tests on time × treatment interaction effects to assess whether the four test meals gave rise to curves of a different shape. If the test resulted in a *P* value <0.05, we tested differences between the meals for each time point. If there were no statistically significant differences in shapes, we tested overall differences between the meals based on the above model without the time × treatment interaction.

To assess summary measures such as iAUC, we fitted a model accounting for the differences in the means of the 10 subjects and the four occasions, and the main effects of treatment. Transformed data were used whenever residual plots showed incompatibility with normality and homogeneity assumptions. We performed an *F* test to see whether there were differences between the meals. If this test resulted in a *P* value <0.05, pairwise comparisons among the meals were conducted.

A Benjamini–Hochberg correction was applied on *P* values from all partial tests to correct for multiple comparisons, resulting in a set of differences in which at most 5 % were falsely selected (*P* < 0.0052 for partial tests was considered significant). The analyses were performed with the software package GenStat, release 13. The within-subject relationship (correlation) between variables was tested by regression analysis according to the method of Bland and Altman [35]. Test meal differences (e.g., density, porosity) were assessed using a Student’s *t* test; a *P* value <0.05 was considered significant. These analyses were performed using SPSS 20.0 for Windows (SPSS Inc., Chicago).

## Results

### Characterization of test meals

The breads (CB and KB, respectively) were comparable in amount of starch (36.2 and 36.4 %), dietary fiber (7.6 and 8.3 %), and moisture content (38.6 and 37.8 %). In vitro quantification of starch fractions showed similar digestive characteristics for CB and KB when a sample from the total bread was analyzed (Table 1).

**Table 1:** In vitro quantification of starch fractions in the test meals

Starch fraction	Test meal/portion			
	CB total	KB total	KB kernel	KB crumb
%				
G_T_	100	100	100	100
G_RA_	78.8	76.4	65.5	67.8
G_SA_	11.8	13.6	15.9	16.3
G_TA_	90.6	90.0	81.4	84.1
RS	8.4	9.0	16.7	14.3

*CB* control bread, *G*_*RA*_ rapidly available glucose (20 min), *G*_*SA*_ slowly available glucose (20–120 min), *G*_*T*_ total glucose (indicated as 100 %), *G*_*TA*_ total available glucose (120 min), *KB* kernel bread, *RS* resistant starch

**Fig. 2:**
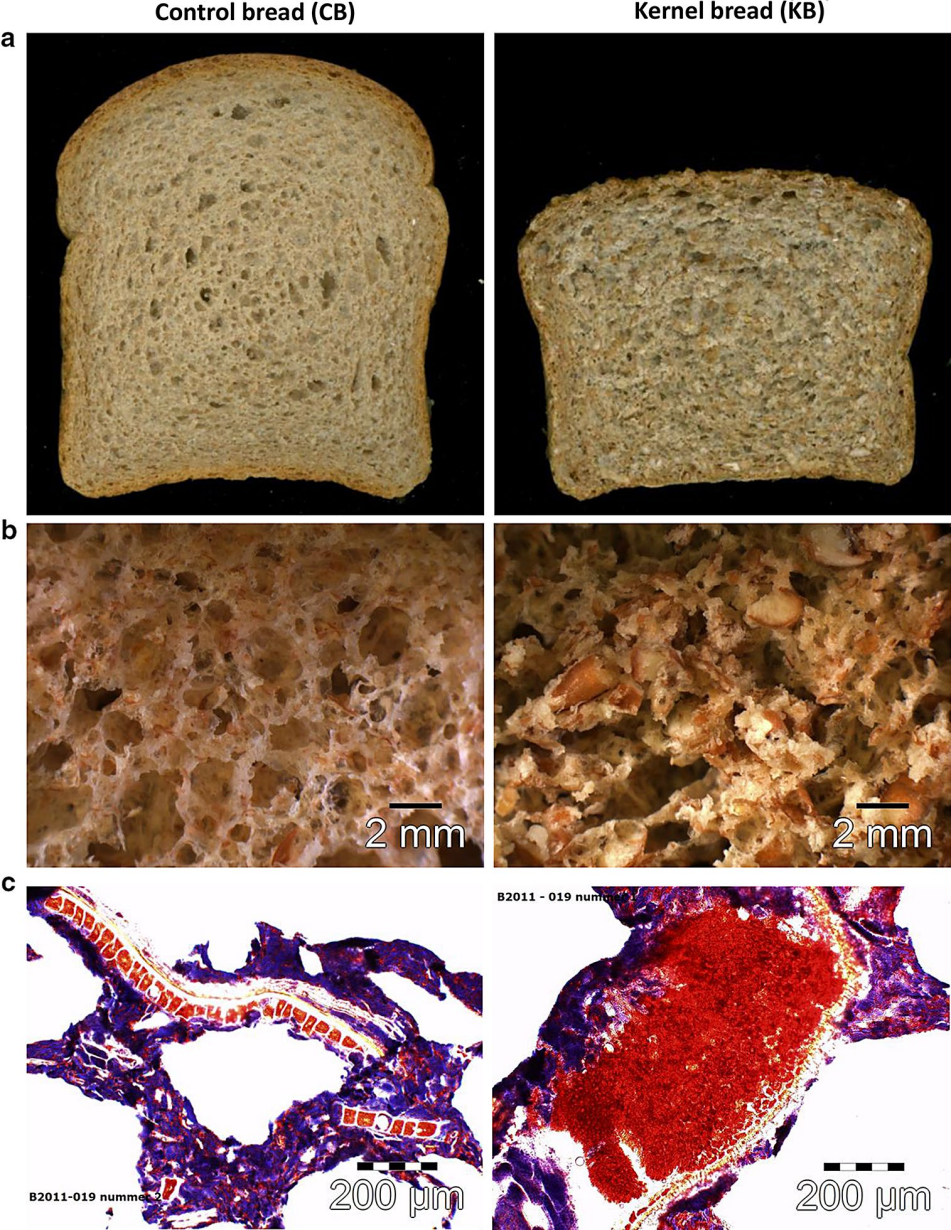
Pictures of ^13^C-enriched control and kernel bread: **a** overview; **b** stereo microscopy; **c** light microscopy with starch (lugol; *blue*) and protein (Ponceau 2R; *red*) staining

A main difference between the two breads (Fig. 2a) was their density; CB had a density of 0.29 ± 0.007 g/mL compared to 0.41 ± 0.003 g/mL for KB (*P* < 0.05). The difference in density was clearly visible in the product structure (Fig. 2b). In the case of KB, 85 % of the wheat material consisted of broken kernels. The majority (72.6 %) of the broken kernels had a particle size between 1680 and 2800 µm as measured by pan sieving. Both visual evaluation and microscopy confirmed that the KB structure consisted of broken wheat kernels with a particle size of 1 to several mm (Fig. 2b). Light microscopy with starch (lugol; blue) and protein staining (Ponceau 2R; red) (Fig. 2c) showed that CB had a porous structure and the thin cell walls were dominated by a continuous phase of starch granules in blue. In this matrix bran particles were clearly visible as clusters of red aleurone cells aligned on the outer side by pericarp layers. An apparent difference was the presence of broken wheat kernels in which no starch granules (blue) were observed (Fig. 2c). Apparently, the matrix was governed by proteins (in red) embedding the starch granules.

A description of the porosity measurements of both breads using XRT can be found in Online Resource 1.

### Postprandial glucose and insulin response

**Fig. 3:**
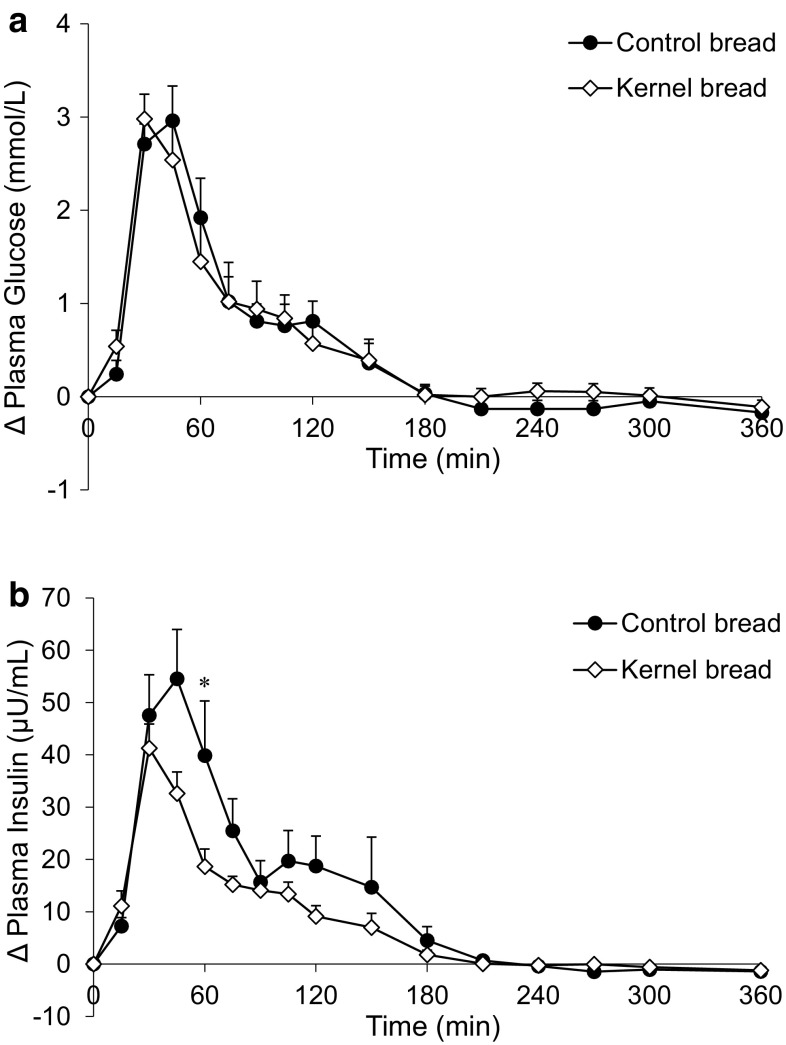
Mean (±SEM) changes from baseline in **a** plasma glucose concentrations and **b** plasma insulin concentrations, after ingestion of 138 g ^13^C-enriched control bread (*filled circle*) and 137 g ^13^C-enriched kernel bread (*open diamond*) in healthy men (*n* = 10). There was no significant time × treatment interaction for glucose (*P* = 0.1773), but there was for insulin (*P* < 0.0001). *Asterisk* significantly different between treatments per time point (after Benjamini–Hochberg correction)

Postprandial glucose concentrations were similar after the consumption of CB and KB (Fig. 3a; Table 2). The insulin response was lower after KB consumption compared to CB at *t* = 60 min (*P* = 0.002) (Fig. 3b), which resulted in a 31 % smaller iAUC (0–2 h) compared with CB intake (NS, *P* = 0.011).

**Table 2:** Indices reflecting the metabolic response after ingestion of 138 g ^13^C-enriched control bread and 137 g ^13^C-enriched kernel bread in healthy men

	Fasting values	Peak values	Time to peak (min)	iAUC (0–2 h)	iAUC (0–6 h)
Glucose (mmol/L)					
CB	5.1 ± 0.1	8.3 ± 0.3	40.5 ± 3.2	163.2 ± 20.5	197.0 ± 23.4
KB	5.0 ± 0.1	8.2 ± 0.3	34.5 ± 2.3	159.4 ± 30.1	195.4 ± 35.4
Insulin (µU/mL)					
CB	4.9 ± 0.5	69.9 ± 10.5	43.5 ± 3.5	3290.9 ± 528.8	4193.0 ± 936.6
KB	5.0 ± 0.5	48.7 ± 4.5	36.0 ± 2.4	2262.1 ± 205.4	2707.6 ± 292.9
Glucagon (pmol/L)					
CB	8.3 ± 1.0	15.8 ± 1.7	175.5 ± 33.4	272.9 ± 84.6	980.5 ± 163.4
KB	10.4 ± 1.2	16.8 ± 2.3	136.5 ± 38.2	207.1 ± 89.4	706.8 ± 199.9
GIP (pmol/L)					
CB	9.2 ± 1.8	84.9 ± 10.5	93.0 ± 13.6	4904.6 ± 426.7	9089.2 ± 1137.8
KB	12.4 ± 1.6	74.5 ± 11.0	84.0 ± 13.5	4494.8 ± 743.1	7169.4 ± 989.0
GLP-1 (pmol/L)					
CB	15.5 ± 1.4	30.9 ± 2.1	86.3 ± 15.7	949.9 ± 95.0	2019.9 ± 183.0
KB	18.3 ± 1.6	30.2 ± 1.8	76.5 ± 16.9	594.7 ± 102.4^a^	1167.0 ± 295.3^a^
RaT (mg/kg min)					
CB	2.0 ± 0.1	6.5 ± 0.3	57.0 ± 14.5	39.1 ± 1.4	88.1 ± 1.6
KB	2.0 ± 0.0	6.8 ± 0.3	36.0 ± 6.0	38.2 ± 1.1	85.1 ± 1.4
RaE (mg/kg min)					
CB	0 ± 0	4.9 ± 0.2	66.0 ± 14.9	49.7 ± 1.6	95.9 ± 4.7
KB	0 ± 0	4.9 ± 0.2	42.0 ± 12.0	46.9 ± 1.7	89.1 ± 1.8
EGP (mg/kg min)^b^					
CB	2.0 ± 0.1	0.9 ± 0.2	111.0 ± 12.1	43.4 ± 9.3	157.8 ± 26.7
KB	2.0 ± 0.0	0.9 ± 0.2	117.0 ± 23.7	40.8 ± 8.8	151.4 ± 24.3
GCR (mL/kg min)					
CB	2.5 ± 0.1	6.4 ± 0.2	100.5 ± 12.5	15.6 ± 1.5	32.7 ± 1.7
KB	2.5 ± 0.1	6.1 ± 0.3	85.5 ± 12.3	15.5 ± 1.9	30.9 ± 2.9
^13^CO_2_ (% dose/h)					
CB	0 ± 0	7.7 ± 0.2	228.0 ± 9.2	4.3 ± 0.3	31.7 ± 0.7
KB	0 ± 0	7.2 ± 0.2^a^	237.0 ± 7.0	4.2 ± 0.2	30.0 ± 0.8
CCK (pmol/L)					
CB	0.5 ± 0.1	2.6 ± 0.3	115.5 ± 10.5	142.4 ± 5.7	295.0 ± 26.4
KB	0.6 ± 0.1	2.2 ± 0.1	126.0 ± 14.0	132.0 ± 13.6	243.0 ± 26.2
Total BA (µmol/L)					
CB	2.1 ± 0.4	4.3 ± 0.7	61.5 ± 19.7	93.0 ± 23.3	171.2 ± 40.9
KB	2.1 ± 0.6	4.2 ± 0.7	63.0 ± 21.1	80.3 ± 18.1	139.8 ± 42.8
Conjugated BA (µmol/L)					
CB	1.2 ± 0.3	3.6 ± 0.6	63.0 ± 19.3	110.8 ± 21.3	216.2 ± 43.4
KB	1.0 ± 0.3	3.3 ± 0.4	64.5 ± 20.7	108.7 ± 24.9	204.5 ± 43.8
Unconjugated BA (µmol/L)^b^					
CB	0.9 ± 0.3	0.2 ± 0.1	211.5 ± 38.0	47.7 ± 26.9	185.2 ± 96.9
KB	1.1 ± 0.5	0.2 ± 0.0	257.3 ± 41.3	60.2 ± 40.5	260.2 ± 160.9

Values are mean ± SEM, *n* = 10

*BA* bile acid, *CB* control bread, *CCK* cholecystokinin, *EGP* endogenous glucose production, *GCR* glucose clearance rate, *GIP* glucose-dependent insulinotropic polypeptide, *GLP-1* glucagon-like peptide-1, *iAUC* incremental area under the curve, *KB* kernel bread, *RaE* rate of appearance of exogenous glucose, *RaT* rate of appearance of total glucose

^a^Significantly different from control bread

^b^Because EGP and unconjugated BAs were suppressed after the test meals, the nadir values and time to nadir are presented. Also, the area beneath baseline (dAUC) was calculated using mirrored data

### Glucose kinetics

**Fig. 4:**
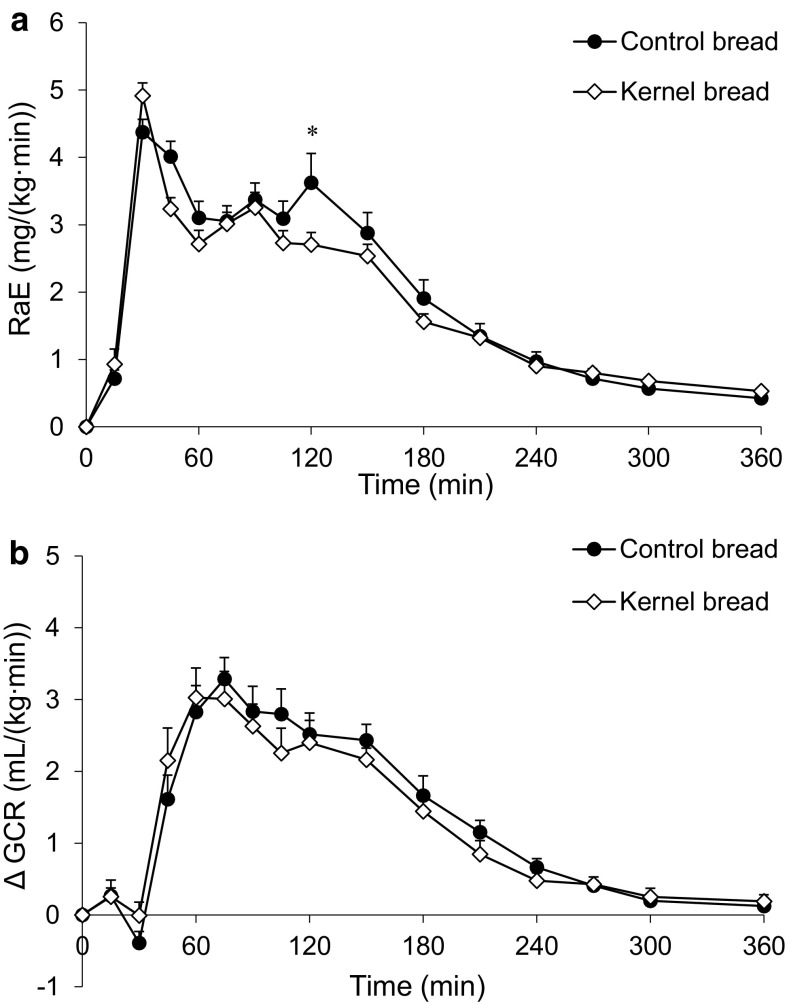
Mean (±SEM) of **a** RaE and **b** changes from baseline in GCR, after ingestion of 138 g ^13^C-enriched control bread (*filled circle*) and 137 g ^13^C-enriched kernel bread (*open diamond*) in healthy men (*n* = 10). There was a significant time × treatment interaction for RaE (*P* < 0.0001) and GCR (*P* = 0.0002). *Asterisk* significantly different between treatments per time point (after Benjamini–Hochberg correction). *RaE* rate of appearance of exogenous glucose, *GCR* glucose clearance rate

The RaE was similar after CB and KB consumption (Fig. 4a), except for *t* = 120 min, where RaE was higher after CB intake (*P* = 0.005). EGP was not significantly different after consumption of CB and KB. The rate at which glucose was cleared from the circulation (GCR) was also similar (Fig. 4b; Table 2).

### Postprandial incretin, glucagon, and CCK response

**Fig. 5:**
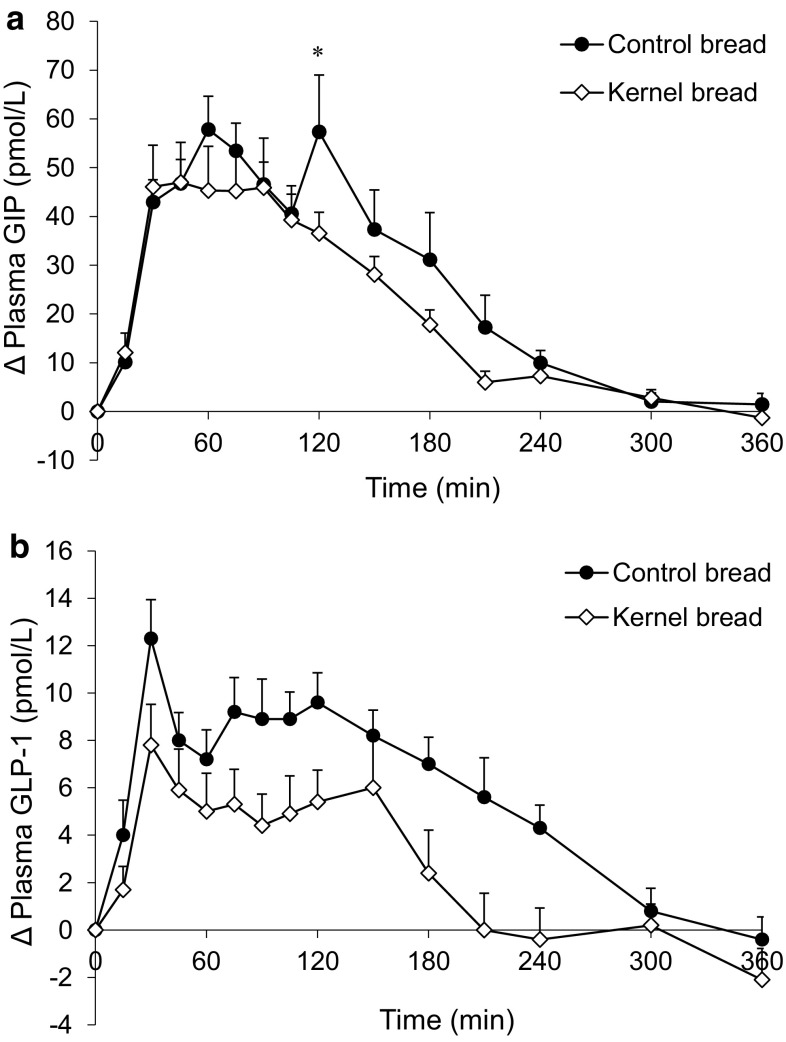
Mean (±SEM) changes from baseline in **a** plasma GIP concentrations and **b** plasma GLP-1 concentrations, after ingestion of 138 g ^13^C-enriched control bread (*filled circle*) and 137 g ^13^C-enriched kernel bread (*open diamond*) in healthy men (*n* = 10). There was a significant time × treatment interaction for GIP (*P* = 0.036), but not for GLP-1 (*P* = 0.073). *Asterisk* significantly different between treatments per time point (after Benjamini–Hochberg correction). *GIP* glucose-dependent insulinotropic polypeptide, *GLP-1* glucagon-like peptide-1

The average postprandial GIP response was somewhat higher after CB compared with KB intake (Fig. 5a; Table 2), but a significant difference was only found at *t* = 120 min (*P* < 0.005). The average GLP-1 response was higher after CB compared to KB consumption at each time point (Fig. 5b), resulting in a difference in the 0–2 and 0–6 h iAUC (Table 2, *P* < 0.005). Differences between time points could, however, not be determined, as time × treatment interaction failed to reach significance (*P* = 0.073).

The postprandial glucagon response appeared higher after CB consumption, but no partial test (CB–KB) could be performed for iAUC. Also, differences between meals at individual time points could not be tested, as there was no time×treatment interaction (*P* = 0.9991).

**Fig. 6:**
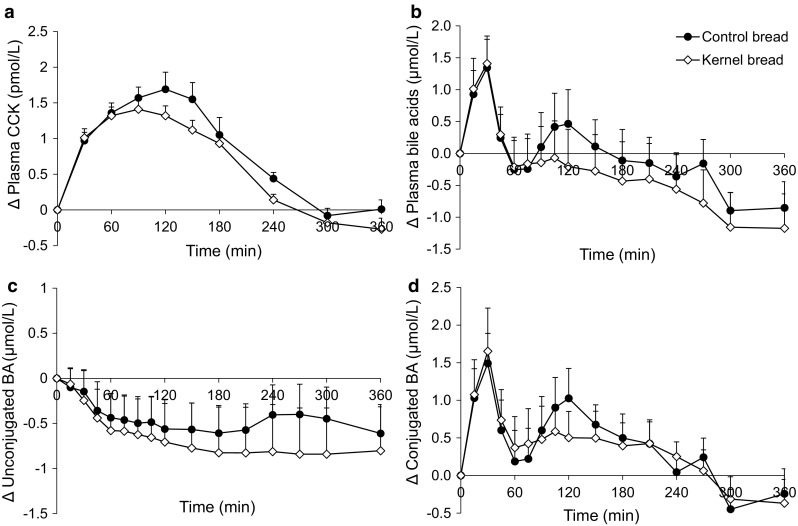
Mean (±SEM) changes from baseline in **a** plasma CCK concentrations **b** total plasma BA concentrations **c** unconjugated BA concentrations and **d** conjugated BA concentrations, after ingestion of 138 g ^13^C-enriched control bread (*filled circle*) and 137 g ^13^C-enriched kernel bread (*open diamond*) in healthy men (*n* = 10). There were no significant time × treatment interactions. *CCK* cholecystokinin, *BA* bile acid

There was a slight increase in CCK concentrations in response to the test meals, which seemed somewhat lower after the KB from 90 min postprandial (Fig. 6a; Table 2), although differences were not significant (iAUC 0–6 h, *P* = 0.03).

### Postprandial bile acid response

The postprandial pattern of glycine and taurine conjugated BAs in plasma was intra-individually identical, with the highest concentrations for GCDCA, followed by either GDCA or GCA. The unconjugated BAs (primary and secondary) also responded in the same way within each person per test period. Therefore, by summing up concentrations, individual BAs were grouped as conjugated and unconjugated BAs, and together they formed the total BA response (Fig. 6b). Unconjugated BAs in plasma showed a decrease after test meal consumption (Fig. 6c), whereas the conjugated BAs increased postprandial and peaked around *t* = 30 min (Fig. 6d). After CB consumption, conjugated BAs showed a second peak around *t* = 120 min. Due to the great inter-individual variation in postprandial responses, *F* tests did not result in significant *P* values, so differences between CB and KB could not be tested.

### ^13^CO_2_ excretion in breath

^13^CO_2_ excretion in breath, reflecting the rate of oxidation of the ^13^C-labeled substrate, was higher after CB consumption at *t* = 210 min (*P* = 0.0014). The time to peak was not different after the meals, but the peak value was significantly higher after CB consumption (*P* = 0.005) (Table 2).

### Correlations

Correlations between several variables (all time points, 0–6 h; *P* < 0.05) were determined (Table 3). RaE and GIP were very well correlated, especially for CB (CB, *r* = 0.84; KB, *r* = 0.71). RaE and GLP-1 showed a moderate correlation (CB, *r* = 0.70; KB, *r* = 0.58). The correlation between GLP-1 and conjugated BAs was moderate for CB (*r* = 0.60) and lower for KB (*r* = 0.39). Total BAs with GLP-1 showed similar correlation coefficients (CB, *r* = 0.53; KB, *r* = 0.43).

**Table 3:** Correlation coefficients relating the glucose, insulin, GIP, GLP-1, conjugated and total BA concentrations, and RaE after ingestion of 138 g ^13^C-enriched CB and 137 g ^13^C-enriched KB

	Glucose		Insulin		GIP		GLP-1		RaE		BA con		BA tot	
	CB	KB	CB	KB	CB	KB	CB	KB	CB	KB	CB	KB	CB	KB
Glucose														
Insulin	0.86	0.83												
GIP	0.57	0.56	0.55	0.63										
GLP-1	0.41	0.43	0.41	0.46	0.59	0.69								
RaE	0.73	0.71	0.66	0.76	0.84	0.71	0.70	0.58						
BA con	0.21	0.24	0.18	0.41	0.34	0.27	0.60	0.39	0.41	0.38				
BA tot	0.23	0.25	0.19	0.39	0.26	0.25	0.53	0.43	0.35	0.27	0.94	0.89		

The within-subject relationship (correlation) between variables was tested by regression analysis according to the method of Bland and Altman [35], *P* < 0.05

*BA con* conjugated bile acids, *BA tot* total bile acids, *CB* control bread, *GIP* glucose-dependent insulinotropic polypeptide, *GLP-1* glucagon-like peptide-1, *KB* kernel bread, *RaE* rate of appearance of exogenous glucose

### Rated appetite, discomfort, and liking of test meal

The subjective sensation of appetite (hunger), as determined hourly using a VAS, was similar after consumption of CB and KB (data not shown). Occasional mild complaints about flatulence were not meal type related. The liking of the test meal was rated by using a VAS (0 = not tasty, 100 = very tasty). The resulting scores (CB = 57, KB = 33) indicate that the KB was not well appreciated by the subjects.

## Discussion

This paper describes the glucose kinetics and metabolic effects in healthy men after consumption of control bread (CB) and 85 % broken kernel bread (KB). We expected that consumption of KB would result in a low glycemic response due to slower digestion of starch in the larger wheat kernel particles [17, 18]. However, the postprandial glycemic response did not differ after consumption of our breads. Although a similar glycemic response does not exclude a difference in in vivo starch digestibility due to possible differences in GCR [33, 34] or EGP, the present study found no pronounced differences in glucose kinetics. The RaE, reflecting intestinal glucose uptake, as well as the GCR and EGP, was similar after KB compared to CB. However, a clear difference in GLP-1 responses was observed after consumption of the test meals.

The use of intact grain kernels in bread formulations has been reported to reduce the glucose response by limiting starch gelatinization and forming a physical barrier for α-amylase, reducing amylolysis [36]. For instance, the incorporation of 80 % preboiled whole wheat kernels [37, 38] showed the expected lowering effect of incorporated kernels on the glycemic response. However, the use of intact kernels per se does not necessarily induce a low GI, as incorporation of oat kernels showed less effect on glycemia [38]. Considering the palatability of the breads, we chose to incorporate broken wheat kernels and not intact kernels in KB, because the replacement of flour (50 and 75 % [18]) with cracked wheat was also previously reported to result in a reduction in GI. The broken kernels were soaked overnight to prevent difficulties in chewing. Even though the broken kernels in KB were still clearly visible, during preparation of the dough the soaked kernels were thoroughly kneaded together with other ingredients, which might have further destructured the kernels, thus increasing starch accessibility to amylolysis. Breads were extensively characterized to document the impact of addition of broken kernels. The porosity, the average size of air cell diameter, and air cell distribution were not significantly different between both breads. However, KB did have a higher density, which in itself is a characteristic that could give rise to a lower glycemic response [39]. Moreover, the average air cell wall thickness of KB was significantly increased compared to CB, but these differences were apparently not large enough to evoke a difference in glucose response or kinetics.

Despite the similarities in the glycemic response, the GLP-1 response was much lower after KB consumption compared to CB (iAUC, *P* < 0.005). Nutrient ingestion is the main stimulus to L cells, which are described as an open type cell, enabling direct stimulation and release of GLP-1 by luminal contact [40]. Although several mechanisms have been proposed with respect to glucose sensing, in the early postprandial phase GLP-1 secretion seems to mainly involve the SGLT-1 glucose transporter in L cells, similar to GIP secretion from K cells [41, 42]. In the present study, glucose transport across the intestinal membrane is reflected by the RaE, which correlated well with GLP-1 concentrations after the CB (*r* = 0.70, *P* < 0.01), but more moderately after the KB (*r* = 0.58, *P* < 0.01). However, the correlations of RaE with GIP (CB: *r* = 0.84 and KB: *r* = 0.71, *P* < 0.01), which are in accordance with our previous studies [31, 34], were stronger. The involvement of additional factors in stimulating GLP-1 release in the present study is thus likely.

We expected that due to the larger particle size in KB, more starch would reach the more abundant distal L cells, resulting in an increased late postprandial GLP-1 response. This was, for instance, seen in a study with slowly digestible starch, together with a prolonged influx of glucose [31]. However, the GLP-1 response was prolonged after CB, but not after KB consumption, even though intestinal glucose uptake did not differ between our breads.

Moreover, with inhibition of SGLT-1 and in SGLT-1-/-mice, a late prolonged increase in GLP-1 after a glucose load was observed [43], besides the decreased GIP and GLP-1 responses in the early postprandial phase [41, 42]. This indicates that the late postprandial GLP-1 response might not require glucose absorption via SGLT-1 [43]. Because L cells are densely distributed in the distal small intestine and colon, it might be that only the presence of still unabsorbed carbohydrates is important in stimulating GLP-1 in the late postprandial phase, for instance via other glucose-sensing mechanisms.

A prolonged postprandial GLP-1 response was also observed when sucrose digestion was delayed by acarbose [44, 45], which was linked to a simultaneous increase in breath hydrogen after 60–120 min, related to fermentation of sucrose. Another explanation for GLP-1 secretion in the late postprandial phase is therefore the involvement of short chain fatty acids (SCFAs), produced during fermentation of unabsorbed carbohydrates or fiber by the intestinal microbiota, and known to stimulate GLP-1 secretion as well [46]. Although fermentation of solid food may be expected in a later phase, in a previous study using ^13^C-labeled barley, ^13^C-labeled SCFAs in plasma were detected within 3 h after ingestion [23]. Increased SCFA formation after CB may be expected due to the presence of finer wheat bran [47].

In addition to nutrients and SCFAs, BAs are also able to potentiate GLP-1 release via activation of the BA receptor TGR5 [13, 14, 48]. Besides in vitro and animal data, in healthy humans, jejunal infusion with TCA and glucose increased GLP-1 concentrations [49]. In our study, conjugated bile acids in plasma increased, whereas the unconjugated BAs decreased after bread consumption. These observations are in agreement with those of others made after an OGTT [50, 51]. However, to the best of our knowledge, so far the differences in BA responses after various starchy food products have not yet been investigated. After KB consumption, the total BA response tended to be lower compared to CB (from *t* = 90 min), but differences were not significant and large inter-individual variations were observed. CCK, involved in regulating gall bladder contraction and thus BA release, showed only a slight increase in response to the test meals, which also tended to be somewhat lower for KB between 90 and 180 min postprandial. However, an apparent difference between CCK and BA response patterns was the peak in BA concentration during the first 60 min, which was not detected in the CCK response in the present study.

We found a moderate correlation between GLP-1 and total BAs (CB *r* = 0.53, KB *r* = 0.43) and GLP-1 and conjugated BAs (CB *r* = 0.60, KB *r* = 0.39), indicating that there may be a relationship between the BA and GLP-1 response. In agreement, another study found a correlation of GLP-1 with mainly glycine-conjugated BAs and total BAs after ingestion of a mixed meal [52]. The correlation coefficients for KB were clearly lower compared to CB, and as mentioned earlier, lower values for KB were also observed when correlating RaE with GIP and GLP-1. Because the interactions are likely to happen in the intestine, it might be that the more preserved kernel structure in KB resulted in less accurate glucose-sensing or interfered in BA binding by the K and/or L cells.

It should also be kept in mind that the BAs measured in the systemic circulation do not necessarily directly reflect what happens in the intestine as, for instance, reabsorption of BAs might be influenced by food components. Fiber was found to bind BAs to a certain extent [53], and with respect to bran, especially more finely milled wheat bran was suggested to have higher BA binding capacity [54]. Thus, the finer wheat bran fiber in CB could have bound more BAs, preventing reabsorption and resulting in higher exposure of the L cells to BAs in the distal small intestine and colon, thereby contributing to the higher late GLP-1 response after CB. In support of this, it was shown in rodents that BA sequestrant administration could increase fecal BAs, stimulate TGR5 on L cells located in the colon, and increase postprandial GLP-1 concentrations [55]. Moreover, rectal infusion of TCA in obese T2DM patients resulted in a pronounced, dose-dependent GLP-1 response [56].

More research is necessary to understand the complex regulation of postprandial GLP-1 stimulation and the possible role of BAs. Therapies that increase BAs (or mimetics) in the distal bowel as a means of increasing endogenous GLP-1 concentrations have been suggested as novel treatments of T2DM and obesity [56]. Several other approaches to increase GLP-1 action are being intensively studied for their potential therapeutic use. Furthermore, because of the beneficial effects in T2DM patients, treatment with GLP-1 analogs was also proposed for people with prediabetes to prevent progression to T2DM [57, 58]. Pharmaceutical intervention might, however, have downsides, and increasing endogenous GLP-1 concentrations by nutritional means would therefore be interesting.

Some potential limitations of this study should be considered. The breads were frozen after baking, to keep them fresh during the whole study period (4-week). Differential effects on digestibility of starch in the control or kernel bread cannot be excluded. Furthermore, we did not measure the protein content of the products. Protein can affect the blood glucose response, by influencing the insulin response independent of carbohydrates. However, we expected the protein content to be very similar, as both breads were made from the same wheat type and also had the same starch content.

To conclude, the substitution of 85 % wheat flour by broken kernels in bread did not result in any difference in glucose response and kinetics, but did elicit a pronounced difference in GLP-1 responses. Because of the complex regulation of GLP-1 release, several factors could simultaneously play a role after consumption of food products such as bread. Plasma bile acids showed a pronounced response after the breads, which might play a role in GLP-1 stimulation.

Our findings show that the glycemic response is not the only parameter which can determine health effects of starchy foods. The GLP-1 response is apparently largely independent from glucose kinetics and might be influenced by other characteristics of starchy food products. Therefore, our finding shows that bread processing technology can influence metabolic response beyond glycemia, which offers significant opportunities.

## Electronic supplementary material

Below is the link to the electronic supplementary material.

Supplementary material 1 (PDF 743 kb)

Supplementary material 2 (PDF 71 kb)
