# Supplementary material for: Difference in postprandial GLP-1 response despite similar glucose kinetics after consumption of wheat breads with different particle size in healthy men
Source: Eur J Nutr. 2016 Feb 8;56(3):1063–76. doi: 10.1007/s00394-016-1156-6 (PMC5346412; doi:10.1007/s00394-016-1156-6)
Supplement: Supplementary file 1 — Supplementary material 1 (PDF 743 kb) [file 394_2016_1156_MOESM1_ESM.pdf]

Online Resource 1

Difference in postprandial GLP-1 response despite similar glucose kinetics after consumption of wheat breads with different particle size in healthy men

European Journal of Nutrition

Coby Eelderink*, Martijn W. J. Noort, Nesli Sozer, Martijn Koehorst, Jens J. Holst, Carolyn F. Deacon, Jens F. Rehfeld, Kaisa Poutanen, Roel J. Vonk, Lizette Oudhuis, Marion G. Priebe

*Corresponding author: C. Eelderink, UMCG, The Netherlands. Email: c.eelderink@umcg.nl

Bread characterization – microscopy and X-ray microtomography

Methods - Microscopy

Stereomicroscopy was performed without sample preparation with an Olympus SZX-9 microscope connected to a DP-50 digital camera (settings Red 1.22; Green 1.22 and Blue 1.22). Light microscopy was performed with an Olympus BH-2 light microscope and the same camera type and settings. Sections of 10 μm thickness were cut in a cryostat-microtome at -26°C and applied to gelatin/glycerol coated slides. Sections were stained for 1 min with Lugol (KI-I2) to color the starch (blue) and for 10 minutes with 0.05% Ponceau 2R solution in 50% glycerol in water to color the protein (red). The excess of stain was rinsed with a minimum of water and sections were covered with glycerol.

Methods - X-ray microtomography

To determine bread porosity, samples were scanned using a desktop X-ray microtomography (XRT) system (Model 1172, SkyScan, Aartselaar, Belgium) consisting of an X-ray tube, an X-ray detector and a CCD camera. The X-ray tube was operated at a voltage of 40 kV/250 µA to obtain optimum contrast between void (air cells) and matter (cell walls) according to a modified method [1]. A 12-bit cooled CCD camera (512 x 1024 pixels) was used to collect

the X-ray data. Samples were rotated by a total of 180° during the scanning process with a pixel size of 24.31 µm to obtain optimum resolution, which gave a total scanning time of 18 min. The initial X-ray radiographs or raw images were obtained at every 0.7° of rotation. In order to avoid water migration during analysis, samples were sealed in stretch film. Samples were scanned in triplicate. After scanning, radiographs were loaded into NRecon reconstruction software (version 1.6.6). The software combined the images graphically into a 3-D object from which 2-D cross sectional images were taken. Before the reconstruction, the CS rotation feature was used to rotate the sample cross sections, making them parallel to the view window. Beam hardening correction was set to 40% in order to reduce the number of artefacts. Cell walls of the solid matrix appear grey, whereas air cells appear black. The reconstructed 2-D slices were then loaded into CTAn software (version 1.12, Skyscan, Belgium) to obtain the parameters of porosity, air cell wall thickness, and air cell diameter.

Results XRT

Although CB seemed more porous than KB, it was not significantly different (Supplemental Figure 1, Supplemental Table 1). Furthermore, the average air cell diameter was not statistically different between CB and KB (Supplemental Table 1). However, the predominant air cell diameter of CB ranged between 200-1000 µm, compared to 600-1200 µm for KB (Supplemental Figure 2a). Generally, KB had a more homogenous cell diameter distribution (Supplemental Figure 2a). Addition of broken wheat kernels resulted in thicker average cell walls in KB and had a major impact on the volumetric distribution of cell wall thickness. CB had a narrower range of cell wall thickness, mostly around 200 µm, whereas KB had a wider profile where the thickness went as high as 1500 µm (Supplemental Figure 2b) due to the presence of large kernel pieces. Average air cell wall thickness of KB (365 ± 33 µm) was more than double compared to CB (147 ± 11 µm) (Supplemental Table 1, $P < 0.05$).

Figures and tables XRT

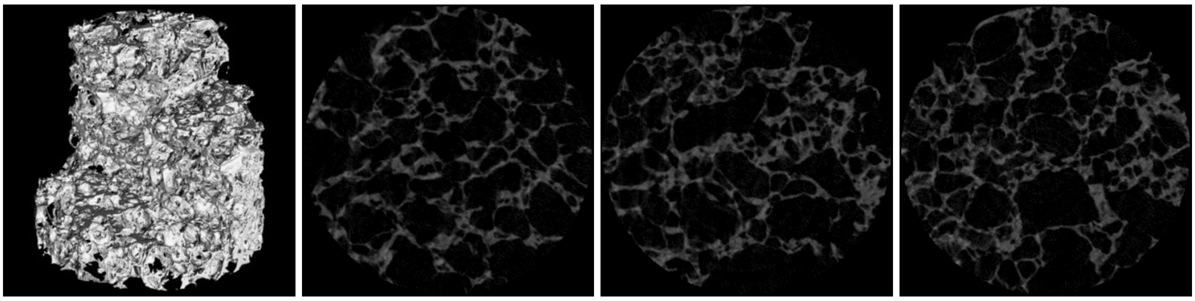

Control bread

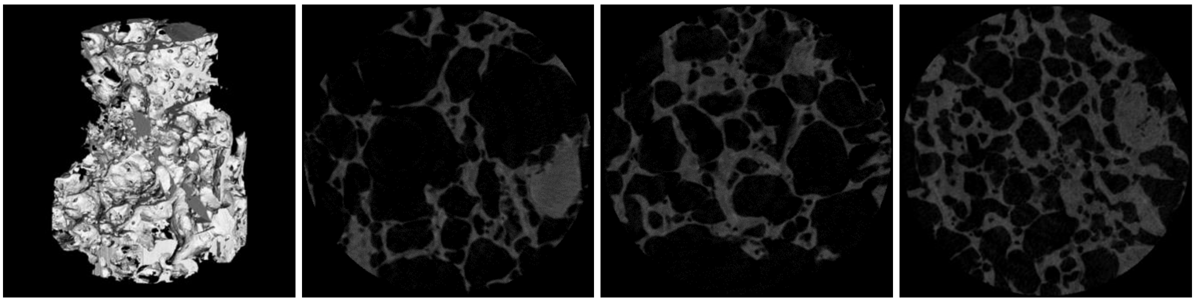

Kernel bread

Supplemental Fig. 1 Reconstructed 3D- and selected representative 2D-images of control and kernel bread using X-ray microtomography.

Supplemental Table 1 Image analysis parameters from 3D XRT data[1]

Sample	Average cell wall thickness (µm)	Average cell diameter (µm)	Porosity (%)
Control bread	143 ± 7^{a}	900 ± 143^{c}	83 ± 3^{d}
Kernel bread	365 ± 33^{b}	1065 ± 266^{c}	73 ± 7^{d}

[1]Values are means ± SD, n = 3. The values marked with different letter in a column are significantly different at 95% ($P < 0.05$) confidence level

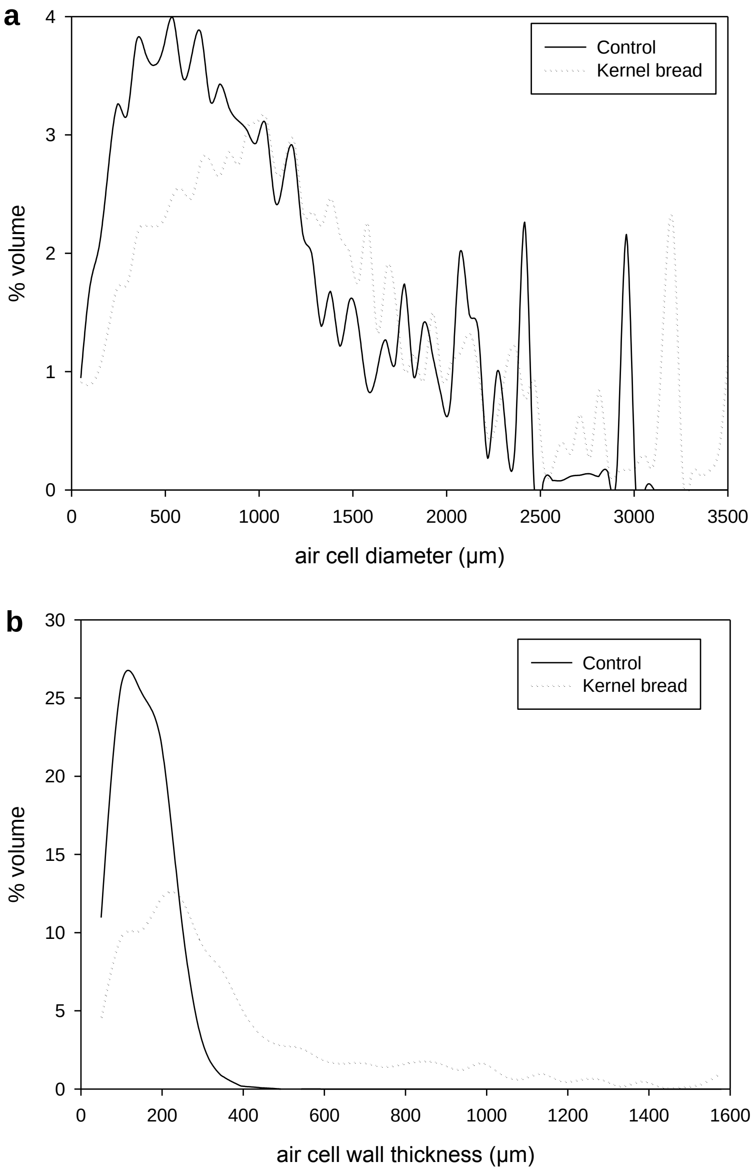

Supplemental Fig. 2 Distribution profiles of (a) air cell diameter and (b) air cell wall thickness of 13C-enriched control bread (—) and kernel bread (·····).

Reference

1. Sozer N, Dogan H, Kokini JL (2011) Textural properties and their correlation to cell structure in porous food materials. J Agric Food Chem 59:1498-1507
