# Supplementary material for: Difference in postprandial GLP-1 response despite similar glucose kinetics after consumption of wheat breads with different particle size in healthy men
Source: Eur J Nutr. 2016 Feb 8;56(3):1063–76. doi: 10.1007/s00394-016-1156-6 (PMC5346412; doi:10.1007/s00394-016-1156-6)
Supplement: Supplementary file 2 — Supplementary material 2 (PDF 71 kb) [file 394_2016_1156_MOESM2_ESM.pdf]

Online Resource 2

Difference in postprandial GLP-1 response despite similar glucose kinetics after consumption of wheat breads with different particle size in healthy men

European Journal of Nutrition

Coby Eelderink*, Martijn W. J. Noort, Nesli Sozer, Martijn Koehorst, Jens J. Holst, Carolyn F. Deacon, Jens F. Rehfeld, Kaisa Poutanen, Roel J. Vonk, Lizette Oudhuis, Marion G. Priebe

*Corresponding author: C. Eelderink, UMCG, The Netherlands. Email: c.eelderink@umcg.nl

Supplemental Methods - Analysis of bile acids

Experimental:

Chemicals and reagents:
Cholic acid (CA), tauro-cholic acid (TCA), glyco-chenodeoxycholic acid (GCDCA), ursodeoxycholic acid (UDCA), tauro-chenodeoxycholic acid (TCDCA),
D4-chenodeoxychol acid (D4-CDCA) and D4-cholic acid (D4-CA) were purchased from Sigma-Aldrich (St. Lous, MO).
Lithocholic acid (LCA), deoxycholic acid (DCA), tauro-deoxycholic acid (TDCA), glyco-deoxycholic acid (GDCA), glyco-lithocholic acid (GLCA), chenodeoxycholic acid (CDCA), glyco-ursodeoxycholic acid (GUDCA), tauro-lithocholic acid (TLCA), glyco-cholic acid (GCA), and tauro-ursodeoxycholic acid (TUDCA) were purchased from Calbiochem.
D4-glyco-chenodeoxycholic acid (D4-GCDCA) and D4-glyco-cholic acid (D4-GCA) were purchased from CDN Isotopes (Pointe-Claire, Quebec, Canada).
D4-tauro-chenodeoxycholic acid (D4-TCDCA) and D4-tauro-cholic acid (D4-TCA) were purchased from Medical Isotopes (Pelham, NH).
HPLC-grade Methanol was purchased from Rathburn Chemicals (Walkerburn, Scotland).
Ammonium acetate and 25% ammonia were purchased from Merck
(Darmstadt, Germany).

Instrumentation:
Two different LC-MS systems were used for the analysis of the plasma samples.
System 1 consists of a SHIMADZU liquid chromatography (LC) system (SHIMADZU, Kyoto, Japan) coupled to an AB SCIEX API-3000 triple quadrupole mass spectrometer with electrospray ionization (ESI) source (AB SCIEX, Framingham, MA).

Electronic Supplementary Material

System 2 consists of an Agilent 1100 liquid chromatography (LC) system (Agilent, Santa Clara, CA) coupled to an AB SCIEX API-3200 triple quadrupole mass spectrometer with electrospray ionization (ESI) source (AB SCIEX, Framingham, MA).
The LC-MS systems are controlled by Analyst 1.6 software.
All chromatographic separations were performed with a XBridge ™ Shield RP18 column (100 mm x 2.1 mm, 3.5 µm) equipped with a XBridge ™ Shield RP18 guard column (10 mm x 2.1 mm, 3.5 µm) (Waters, Milford, MA).

Liquid chromatographic and mass spectrometric conditions:
The mobile phase consisted of 20 mM ammonium acetate, adjusted to pH 8.0 with 25% ammonia (mobile phase A) and methanol (mobile phase B), at a total flow rate of 0.2 ml/min. The gradient profile is shown in Supplemental table 1.

Supplemental table 1 Gradient profile

**Total time (min)**	**Flow rate (ml/min)**	**%A**	**%B**
0.0	0.200	60	40
22.0	0.200	20	80
22.5	0.200	5	95
25.5	0.200	5	95
26.0	0.200	60	40
33.5	0.200	60	40

The injection volume of all samples was 10 µl.
The mass spectrometer parameters, such as gas pressure, voltage, temperature, etc., were optimized by infusing each analyte and the internal standards (IS) separate in a 50% MeOH solution via a Harvard pump 11 standard infusion syringe pump (Harvard Apparatus, South Natick, MA).
All bile acids were detected in negative mode with the mass spectrometer source settings shown in Supplemental table 2 and 3.

Supplemental table 2 Mass spectrometer source settings (system 1-API-3000) period 1 and 2

**Detection mode**	MRM, negative mode
**Resolution Q1**	Unit
**Resolution Q3**	Unit
**Nebulizer gas**	12
**Curtain gas**	10
**CAD gas**	5
**Ion spray voltage**	-3000V
**Temperature**	450 °C
**EP**	-10
**MR pause**	5 msec

Supplemental table 3 Mass spectrometer source settings (system 2-API-3200) period 1 and 2

**Detection mode**	MRM, negative mode
**Resolution Q1**	Unit
**Resolution Q3**	Unit
**Collision gas**	10
**CAD gas**	10
**Ion source gas 1**	25
**Ion source gas 2**	30
**Temperature**	600 ºC
**Ion spray voltage**	-4500V
**MR pause**	5 msec

The multiple reaction monitoring (MRM) transitions for each bile acid and internal standard, as well as their optimum MS parameters such as collision energy (CE), declustering potential (DP), focusing potential (FP), cell exit potential (CXP) and cell entrance potential (CEP) are shown in Supplemental table 4 and 5.

Supplemental table 4 MRM settings system 1 (API-3000)

Component	Q1 mass	Q3 mass	Dwell time (msec)	Declus-tering potential (DP)	Focus-sing potential (FP)	Collision energy (CE)	Cell exit potential (CXP)
UDCA	391.3	391.3	90	-96	-240	-5	-17
CA	407.2	407.2	90	-96	-140	-5	-17
D4-CA	411.2	411.2	90	-96	-140	-5	-17
GUDCA	448.4	74.0	90	-90	-310	-70	-15
GCA	464.2	74.1	90	-100	-240	-76	-13
D4-GCA	468.2	74.1	90	-100	-240	-76	-13
TUDCA	498.4	79.9	90	-101	-300	-102	-10
TCA	514.2	79.9	90	-100	-230	-100	-5
D4-TCA	518.2	79.9	90	-100	-230	-100	5
LCA	375.1	375.1	90	-101	-280	-5	-17
CDCA, DCA	391.3	391.3	90	-96	-240	-5	-17
D4-CDCA	395.3	395.3	90	-96	-240	-5	-17
GLCA	432.1	74.0	90	-101	-280	-68	-5
GCDCA, GDCA	448.4	74.0	90	-90	-310	-70	-15
D4-GCDCA	452.4	74.0	90	-90	-310	-70	-15
TLCA	482.2	79.8	90	-90	-340	-98	-5
TCDCA, TDCA	498.4	79.9	90	-101	-300	-102	-10
D4-TDCA	502.4	79.9	90	-101	-300	-102	-10

Supplemental table 5 MRM settings system 2 (API-3200)

Component	Q1 mass	Q3 mass	Declus-tering potential (DP)	Cell entrance potential (CEP)	Collision energy (CE)	Cell exit potential (CXP)	Entrance Potential (EP)	Dwell time (msec)
UDCA	391.3	391.3	-130	-26	-13	-4	-4	90
CA	407.2	407.2	-28	-21	-14	-4	-12	90
D4-CA	411.2	411.2	-28	-21	-14	-4	-12	90
GUDCA	448.4	74.0	-118	-20	-63	-1	-10	90
GCA	464.2	74.1	-106	-23	-65	-1	-7	90
D4-GCA	468.2	74.1	-106	-23	-65	-1	-7	90
TUDCA	498.4	79.9	-23	-21	-83	-1	-5	90
TCA	514.2	79.9	-39	-25	-121	-1	-4	90
D4-TCA	518.2	79.9	-39	-25	-121	-1	-4	90
LCA	375.1	375.1	-123	-18	-13	-6	-7	90
CDCA	391.3	391.3	-134	-19	-13	-5	-10	90
DCA	391.3	391.3	-115	-19	-15	-5	-6	90
D4-CDCA	395.3	395.3	-134	-19	-13	-5	-40	90
GLCA	432.1	74.0	-100	-22	-58	-2	-4	90
GCDCA	448.4	74.0	-102	-20	-62	-1	-7	90
GDCA	448.4	74.0	-103	-20	-62	-1	-7	90
D4-GCDCA	452.4	74.0	-102	-20	-62	-1	-7	90
TLCA	482.2	79.8	-60	-22	-109	-1	-7	90
TCDCA	498.4	79.9	-146	-23	-111	-1	-7	90
TDCA	498.4	79.9	-153	-22	-115	-2	-7	90
D4-TDCA	502.4	79.9	-153	-22	-115	-2	-7	90

Sample preparation:

For plasma samples, 250 µl of internal standard solution was added to 25 µl plasma, vortexed, and centrifuged at 15.900 x g for 10 min. The supernatant was transferred into a new vial, evaporated under nitrogen at 40 ^{o}C, and reconstituted in 100 µl of 50% methanol. The solution is now filtered with a 0.2 µm centrifugal filter at 2000 x g.

After this step the samples are ready for analysis.
